# The association of sperm *BAX* and *BCL-2* gene expression with reproductive outcome in Oligoasthenoteratozoospermia cases undergoing intracytoplasmic sperm injection: A case-control study

**DOI:** 10.18502/ijrm.v22i6.16797

**Published:** 2024-08-05

**Authors:** Fatemeh Babaei, Maryam Khoshsokhan Muzaffar, Rahil Jannatifar

**Affiliations:** ^1^Biology Department, Islamic Azad University, Qom Branch, Qom, Iran.; ^2^Department of Reproductive Biology, Academic Center for Education, Culture, and Research (ACECR), Qom, Iran.

**Keywords:** BAX, BCL-2, Fertilization, Embryo.

## Abstract

**Background:**

The B-cell lymphoma 2 (*BCL-2*) protein is one of the members of the *BCL-2 *associated X (*BAX*) protein family that acts as an inducer of apoptosis.

**Objective:**

The present study aims to investigate the association between *BAX* and *BCL-2* gene expression with reproductive outcome, in cases undergoing intracytoplasmic sperm injection.

**Materials and Methods:**

In this case-control study, 50 men were divided into healthy fertile and oligoasthenoteratozoospermic infertile men (n = 25/each). They were subjected to history taking, clinical examination, and semen analysis. Expression of *BAX* and *BCL-2* genes were measured using real-time polymerase chain reaction. The DNA fragmentation index was measured using the sperm chromatin dispersion assay technique. Using World Health Organization criteria, sperm parameters were evaluated.

**Results:**

Evaluation of apoptosis-related genes showed that oligoasthenoteratozoospermic significantly increased mRNA expression of *BAX*, and significantly decreased mRNA expression of *BCL-2*, when compared with control. Moreover, the *BAX/BCL-2* ratio was significantly higher in oligoasthenoteratozoospermic compared to the normozoospermic group (p = 0.01). Also, this study showed that the *BAX* and *BCL-2* genes expression had a significant correlation with sperm quality, and DNA fragmentation in the oligoasthenoteratozoospermic group (p = 0.01). The oligoasthenoteratozoospermic men, had a considerably lower proportion of fertilization rate and good-quality embryos at the cleavage stage than the normozoospermic subjects (p = 0.01). A significant correlation was observed between the expression of *BAX* and *BCL-2* genes, fertilization, and embryo quality (p = 0.01).

**Conclusion:**

We concluded that the sperm *BAX/BCL-2* ratio demonstrates a significant correlation with fertilization rate and embryo quality.

## 1. Introduction 

Apoptosis (programmed cell death) is a regulated process leading to the removal of damaged, surplus, or aged cells during normal development, and its deregulation causes many disorders (1). In the absence of specific cell surface receptors, factors that can directly penetrate the cell and modulate the apoptotic cascade may activate apoptosis (2). Such factors include: heat shock, ultraviolet radiation, reaction oxidative stress, and some drugs. Today, the presence and activity of apoptosis signals in human sperm in response to various stimuli is widely accepted (3).

There are 2 distinct mechanisms for the initiation of apoptosis: extrinsic pathway or receptor apoptosis and endogenous or mitochondrial apoptosis. Specific mechanisms include the cell DNA fragmentation through chromatin degradation during programmed cell death, a biological mechanism critical for generating appropriate germ cells and maintaining the germ cell-to-Sertoli ratio in the testis (3). In fact, the biochemical feature of apoptosis includes the transfer of phosphatidylserine to the outer plasma membrane, caspase activation, and DNA fragmentation (4). Caspase activity is associated with sperm immaturity, low number, reduced motility, lower fertilization levels (5).

Apoptosis in spermatogenesis is a dynamic process including morphologic differentiation, mitotic divisions of spermatogonia and spermatocytes, and spermatids ending maturation in sperm formation (6). In many types of cells, there are several proteins that play a role in regulating apoptosis. It has been reported that some members of the B-cell lymphoma 2 (*BCL-2*) family (BCL-w, Mcl-7, BCL-xl, BCL-2) are involved in suppressing apoptosis, and some of them (Bad, Bak, BCL-xs, BAX) in promoting apoptosis (7). *BCL-2* protein suppresses apoptosis, while *BAX* protein promotes apoptosis. *BCL-2 *helps maintain mitochondrial membrane integrity by binding it to mitochondrial outer membrane receptors. When cells are exposed to apoptosis-inducing agents, *BAX *is translocated from the cytoplasm to the mitochondrial membrane, causing changes in cell membrane permeability (8). Following these changes, cytochrome C is released and induces apoptosis, which ultimately leads to sperm DNA fragmentation.

The primordial germ cells originate from the epiblast and finally migrate to the gonad. Excess cells produced at this time are killed by apoptosis, which mainly depends on coordination between *BCL-xL* and *BAX* (9). An abrogated initial wave of apoptosis in transgenic mice overexpressing *BCL-2* or *BCL-x *leads to accumulation of spermatogonia and spermatocytes, resulting in infertile animals (10). *BCL*-x-knockout mice have severe defects in male germ cells during development.

The balance between proteins that are involved in the induction of apoptosis and those that suppress apoptosis in the cell affects the regulation of apoptosis in the cell. For example, a high *BAX/BCL* ratio indicates a pro-apoptotic tendency of the cell (11). By increasing the amount of *BAX* and decreasing *BCL-2*, a suitable pre-apoptotic environment is prepared for the cell (12). Today, the use of intracytoplasmic sperm injection (ICSI) is suggested for better selection of sperm in patients with severe male factor infertility (13). Poor seminal parameters, including increased sperm DNA fragmentation, decrease blastocyst production rates following in vitro fertilization, suggesting an effect of sperm on preimplantation embryo development in humans (14). Studies have shown that increasing the amount of apoptosis in sperm decreases the quality of sperm parameters and then increases the fragmentation rate in the embryo (15).

This study aims to explore the association of sperm *BAX* and *BCL-2* gene expression with reproductive outcome in oligoasthenoteratozoospermic cases undergoing ICSI.

## 2. Materials and Methods

### Study design

This study is a case-control method that was randomly sampled among 25 infertile men (oligoasthenoteratozoospermic) undergoing assisted reproductive technology (ART) cycles in the age range of 20–40 yr who were approved by a urologist and referred to the Infertility Research Center, the Academic Center for Education, Culture, and Research (ACECR), Qom, Iran from August 2022-March 2023. Men with total sperm motility < 40% and count < 15 million sperm/ml and normal morphology < 4%, participated as oligoasthenoteratozoospermic according to the analysis by the World Health Organization (WHO) (16). 25 normozoospermic men were included as healthy control in the age range of 20–40 who are healthy according to the analysis of the WHO. Those with smoking and alcohol assumption, varicoceles, systemic diseases, cryptorchidism and external genitalia abnormalities, and hormonal level abnormalities were excluded from this study. Partner's age of patients who are oligoasthenoteratozoospermic was between 25–35 yr, and there were no female disorder factors such as ovulation disorders, tubal factor, endometrial disease, or endocrine abnormalities. Also, participants with a history of alcohol abuse, drug use, or smoking were excluded from the study.

### Sperm analysis

Following 4–5 days of sexual abstinence, the ejaculates were poured into sterile containers, and more than 1 sample was prepared at an interval of 2 wk from each other. The semen examination was based on the WHO instructions (16).

Computer-assisted sperm analysis system (LABOMED, SDC313B, Germany) was used to assess sperm motility (progressive, non-progressive or quiescent). Papanicolaou staining was also used to evaluate the normal morphology of sperms. To check the number of sperm, after appropriate dilution, 100 sperm per slide were measured by Neubauer chamber through a Nikon Eclipse TE2000 microscope.

### *BAX* and *BCL*-2 gene expressions 

Total ribonucleic acid (RNA) was extracted from all sperm samples by total RNA extraction as well as first strand cDNA generation using QuantiTectⓇ, and RNeasy Micro kits (Qiagen, Europe). The NanoDrop spectrophotometer was applied to determine RNA concentration, which was adjusted to 1000 ng/ μ l. The RevertAid First Strand cDNA synthesis kit (Thermo Fisher Scientific Inc. China) was applied to synthesize cDNA on the same day as guided. The reverse transcription was done in 20 μ l reactions (42 C/60 min and then 70 C/5 min) for inactivation of the reverse transcriptase and its product was directly applied in quantitative polymerase chain reaction (PCR) in an independent step to amplify the targets.

### Real-time PCR 

Quantitative real-time PCR was used to evaluate BCL-2 and BAX relative transcript levels using specified primers (Table I). The internal control was the glyceraldehyde-3-phosphate dehydrogenase (*GAPDH*) gene. The QuantiTect SYBR Green RT-PCR kit (Lot No.: 1201416; Applied Biosystems, UK) was used for PCR run on an ABI 7500 RT-PCR system (Applied Biosystems) considering stage 1 (95 C/5 min) and stage 2 (95 C/20 s, 58 C/30 s, 72 C/20 s) for 40 cycles, followed by a melt curve stage (95 C/15 s, 58 C/1 min, and 95 C/15 s). The specimens were run 2 times to decrease the sampling error, and the duplicates' mean value was applied for the next calculations. Specimens minus the reverse transcriptase and no template control were run along with the original specimens. The product length, 2% agarose gel, and a melting curve analysis verified amplicon size and specificity. The ratio = (E target) Δ Ct target (control sample)/(E ref) Δ Ct ref (control-sample) formula was used to calculate the relative transcript levels. The target gene relative transcript levels were calculated using the RT PCR efficiency (E) as well as the threshold cycle difference related to an unfamiliar specimen compared to the control (Ct control-sample). CDNA dilution curves were constructed for each gene to determine the individual real-time PCR efficiencies (E = 10 [- 1/slope]).

### Sperm DNA fragmentation

The terminal deoxynucleotidyl transferase dUTP nick end labeling assay evaluated sperm DNA fragmentation by the in-situ cell death detection kit (Roche, Germany) (17). A fluorescence microscope (BX51, Olympus, Japan) was used to assess 500 sperms for each slide at × 100 magnification. Sperm characterized by redheads had intact DNA, whereas sperm with green heads had fragmented DNA.

### ICSI process, and embryo quality 

A long method of ovulation induction was performed in all the cases' wives. 250 gr of recombinant human chorionic gonadotropin (OVIDREL, hCG, Merck-Serono) was injected to trigger follicular development, followed by the extraction of oocytes (18). Which were obtained from follicular fluid quickly after follicle aspiration. Oocytes with the first polar body morphology were used for ICSI. Then, 24 hr after oocyte retrieval, an injection of normal sperm was done to inseminate the oocyte. Then 16–18 hr after ICSI, 2 pronuclei observed by an Olympus inverted microscope using a Hoffmann modulation contrast system ( × 400) proved fertilization. Embryo quality and cleavage rate were assessed 3 days following ICSI, according to a scoring system (19): grade I, no fragmentation and symmetric blastomeres; grade II, uneven blastomeres and less than 30% fragmentation; and grade III, uneven blastomeres and more than 30% fragmentation. The resulting embryos were cryopreserved. 2 freeze-thaw embryo transfer ICSI cycles were performed in all cases.

**Table 1:** The *BAX* and *BCL-2* and *GAPDH* genes and their product length


**Accession numbers**	**Gene**	**Primer sequence**	**Product size (bp)**
**NM_001291428**	*BAX*	Forward: 5 ' -CGGAACTGATCAGA ATC-3 ' Reverse: 5 ' -GACATCAGTCGCTT CAGTG-3 '	124
**NM_000633**	*BCL-2*	Forward: 5 ' -AGAAAGCAGGAAAC CTGTGG-3 ' Reverse: 5 ' -GATAGCAGCACAGG ATTGGA-3 '	126
**NM_032991**	*GAPDH*	Forward: 5 ' -TGGTATCGTGGAAG GACTCA-3 ' Reverse: 5 ' -CCAGTAGAGGCAGG GATGAT- '	132
*BAX: BCL-2* associated X,* BCL-2*: B-cell lymphoma 2, *GAPDH*: Glyceraldehyde-3-phosphate dehydrogenase			

### Ethical considerations 

The Ethics Committee for Human Subjects Research of Qom Azad University, Qom, Iran accepted this study (Code: IR.IAU.QOM.REC.1401.072). Patients enrolled in the study, and the proband's family gave their written approval.

### Statistical analysis

We used the Statistical Package for Social Sciences (SPSS) version 16 to analyze the data. In addition, variables with a continuous normal distribution were examined using the independent sample *t* test, while the Mann-Whitney U test was used for data that were not normally distributed. P < 0.05 was considered statistically significant. Findings are shown as mean ± SEM.

## 3. Results 

### Clinical and demographic characteristics

No significant differences were observed in the male age (p = 0.40), female age (p = 0.49) duration of marriage (p = 0.96), or duration of infertility (p = 0.95). Also, no significant differences were observed in weight, height, or BMI between the 2 groups as presented in table II.

### Sperm quality

Table III indicate that sperm concentration (p = 0.001), total motility (p = 0.001), progressive motility (p = 0.004), and normal morphology (p = 0.001), between oligoasthenoteratozoospermia, and normozoospermic fertile subjects were significantly different. Also, data were significantly different for DNA fragmentation of sperm between oligoasthenoteratozoospermic, and normozoospermic subjects (p = 0.013). No significant differences were observed in the semen volume between the 2 groups.

### *BAX* and *BCL*-2 gene expression

**Figure 1:**
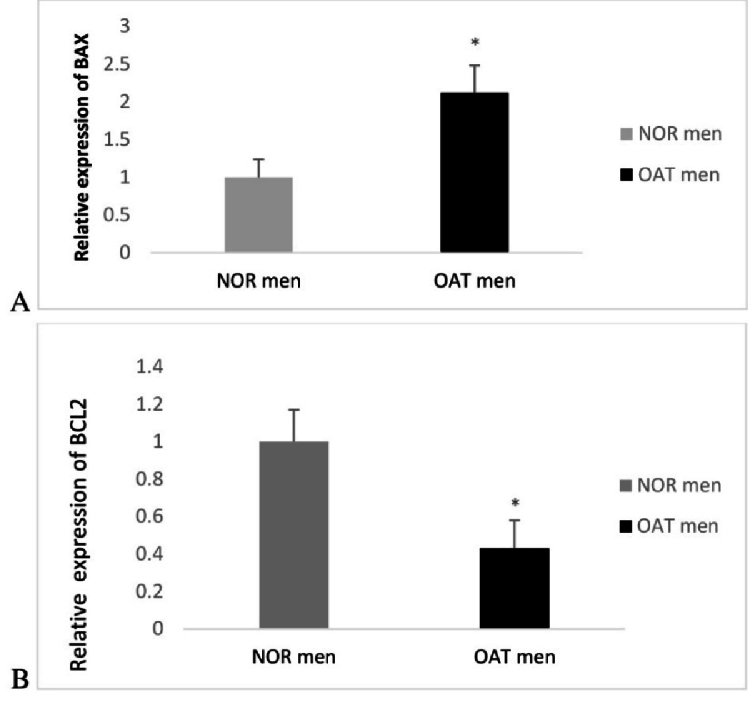
Comparison of *BAX* (A) and *BCL-2 *(B)gene expression in 2 groups. mRNA expression of *BAX *significantly increased as compared with normozoospermic men patients. However, mRNA expression of *BCL-2* significantly decreased in oligoasthenoteratozoospermic as compared with normozoospermic patients. Data are shown as Mean ± SEM. OAT: Oligoasthenoteratozoospermic, NOR: Normozoospermic, *BAX*: *BCL*-2 associated X, *BCL*-2: B-cell lymphoma 2.

Figure 1A, B presents the evaluation of apoptosis-related genes showing that mRNA expression of *BAX* significantly increased, compared to fertile patients (p = 0.012). However, mRNA expression of *BCL-2* significantly decreased in oligoasthenoteratozoospermic as compared with normozoospermic men (p = 0.032). As expected, the *BAX/BCL-2* ratio was significantly higher in oligoasthenoteratozoospermic compared to the normozoospermic group (p = 0.033).

### Correlation of *BAX*, *BCL*-2 with sperm parameters 

The correlations analysis between *BAX* and *BCL-2 *gene expression with sperm parameters and DNA fragmentation (Table IV) shows that there is a significant negative correlation with sperm motility (p = 0.001), sperm count (p = 0.001), and sperm normal forms with *BAX *gene expression (p = 0.001), and whether, there is any significant positive correlation with sperm DNA fragmentation (p = 0.01; Table II). Seminal *BCL-2* demonstrated a significant correlation with sperm count (p = 0.001), sperm motility (p = 0.001), and sperm morphology (p = 0.014) while a negative correlation was observed with sperm DNA fragmentation (p = 0.010; Table IV).

### Fertilization rate and embryo quality after ICSI

As shown in table V, oligoasthenoteratozoospermic men had a considerably lower proportion of fertilization rate and good-quality embryos at the cleavage stage than the normozoospermic subjects. A significant upper fertilization rate was observed in fertile men compared to oligoasthenoteratozoospermic (p = 0.030). Also, the cleavage rate was significantly higher in fertile men compared to oligoasthenoteratozoospermic men (p = 0.024). The mean percentages of embryo quality scores were substantially different between groups (A, B) (p = 0.001) and (p = 0.015), respectively. A significant difference in embryo grade C was observed between normozoospermic and oligoasthenoteratozoospermic men (p = 0.012).

### Correlation of *BAX*, *BCL*-2 with fertilization rate and embryo quality

The Sperm* BAX *gene expression had a significantly negative correlation with fertilization rate (p = 0.036), cleavage rate (p = 0.046), embryo quality (grade A) (p = 0.044) embryo quality (grade B) (p = 0.05), and embryo quality (grade C) (p = 0.043). Whether sperm *BCL-2* gene expression demonstrated a significant positive correlation with fertilization rate (p = 0.041), cleavage rate (p = 0.048), and embryo quality (grade A) (p = 0.041), embryo quality (grade B) (p = 0.034), and embryo quality (grade C) (p = 0.011; Table VI).

**Table 2:** Clinical characteristics of the oligoasthenoteratozoospermic and normozoospermic subjects


**Clinical characteristics**	**Oligoasthenoteratozoospermic men**	**Normozoospermic men**	**P-value**
**Male age (yr)**	37.5 ± 2.3	36.0 ± 1.8	0.40
**Female age (yr)**	29.67 ± 3.35	28.07 ± 3.41	0.49
**The mean duration of marriage (yr)**	8.07 ± 3.96	8.6 ± 3.1	0.96
**The mean duration of infertility (yr)**	6.63 ± 3.62	6.77 ± 3.01	0.95
**Weight (kg)**	85.13 ± 11.9	86.26 ± 10.1	0.99
**Height (cm)**	164.53 ± 5.31	165.46 ± 6.7	0.87
**BMI**	31.68 ± 4.5	31.6 1 ± 4.1	0.85
Data presented as Mean ± SEM. Independent sample *t* test. BMI: Body mass index			

**Table 3:** The results from sperm parameters in oligoasthenoteratozoospermic and normozoospermic subjects


**Sperm parameters**	**Oligoasthenoteratozoospermic men**	**Normozoospermic men**	**P-value**
**Semen volume (ml)**	2.9 ± 0.3	3.0 ± 0.4	0.84
**Sperm concentration (10^6^/ml)**	10.52 ± 3.80	75.06 ± 12.51	0.001*
**TM (%)**	31.42 ± 8.60	70.18 ± 11.21	0.001*
**PM (%)**	20.48 ± 8.57	44.54 ± 10.08	0.001*
**Normal morphology (%)**	95.02 ± 2.16	98.02 ± 1.16	0.001*
**DFI (%)**	16 ± 1.47	9.14 ± 2.46	0.013*
Data presented as Mean ± SEM. Independent sample *t* test. *Statistically significant compared to fertile group (p < 0.05). TM: Total motility, PM: Progressive motility, DFI: DNA fragmentation index			

**Table 4:** Correlations between *BAX* and *BCL-2* mRNA levels with sperm parameters


	***BAX***		***BCL-2***	
**Correlation**	**r**	**P-value**	**r**	**P-value**
	**Concentration**	-0.727*	0.001	0.734*	0.001
**Total motility**	-0.705*	0.001	0.681*	0.001
**Normal forms**	-0.748*	0.001	0.687*	0.014
**DFI**	0.642*	0.01	-793*	0.01
r: Shows the Pearson correlation coefficient. The statistical correlation p < 0.05 was significant. *Correlation is significant at the 0.05 level (2-tailed). *BAX*: *BCL*-2 associated X, *BCL*-2: B-cell lymphoma 2, DFI: DNA fragmentation index				

**Table 5:** Comparing ICSI outcome between oligoasthenoteratozoospermic and normospermic subjects


**Parameters**	**Oligoasthenoteratozoospermic men**	**Normospermic men**	**P-value**
**Fertilization rate (%)**	69.65	87.2*	0.03
**Cleavage rate (%)**	55.8	76.8*	0.02
**Grade A (no fragmentation)**	55.3	76.2**	0.00
**Grade B ( > 20%)**	10.30	14.52*	0.01
**Grade C ( < 20%)**	44.2	23.2*	0.01
Data presented as percentages. *Statistically significant compared to fertile group (p < 0.05). **Statistically significant compared to normospermic group (p < 0.05). ICSI: Intracytoplasmic sperm injection			

**Table 6:** Correlations between *BAX*, *BCL-2* mRNA levels, fertilization, and embryo quality


**Correlation**		***BAX***		***BCL-2***	
		**r**	**P-value**	**r**	**P-value**
**Fertilization**		-0.217*	0.03	0.207*	0.04
**Cleavage**		-0.413*	0.04	0.444*	0.04
**Embryo grade**					
	**A (no fragmentation)**	-0.464*	0.04	0.461*	0.04
	**B ( > 20%)**	-0.342*	0.0	0.351*	0.03
	**C ( < 20%)**	-0.595*	0.04	0.607*	0.01
The statistical correlation p < 0.05 was significant. *Correlation is significant at the 0.05 level (2-tailed), r: Shows the Pearson correlation coefficient. *BAX*: *BCL*-2 associated X, *BCL*-2: B-cell lymphoma 2					

## 4. Discussion

The main aim of this study was to investigate the increase of apoptosis in ART results based on sperm quality and DNA damage level. Our study showed that the mRNA expression of *BAX* were significantly increased in the oligoasthenoteratozoospermic group compared to normozoospermic individuals. Whereas, *BCL-2* mRNA expression was significantly decreased in oligoasthenoteratozoospermic men compared to normozoospermic men. Also, the *BAX/BCL-2* ratio of sperm in infertile men increased significantly compared to fertile men. Sperm *BAX* gene expression had a significant and negative correlation with sperm motility, concentration, and normal shapes, while sperm *BCL-2* gene expression had a positive and significant correlation with these parameters. Also, we detected a significant correlation between *BAX* and *BCL-2* expression with DNA denaturation.

The higher expression of *BAX* compared to *BCL-2* leads to forming pores associated with apoptosis and possibly triggers apoptotic reaction cascades (20). The cascade was initiated by the release of mitochondrial cytochrome-c into the cytosol and interacted with Apaf-1 to generate apoptosomes to recruit and activate procaspase-9 (21). Caspase effectors could destroy several substrates in the cell leading to morphological alterations in apoptosis (22). Activation of caspase-9 in ejaculated sperm occurs in the post-acrosomal stage in mature sperm (23), and the midpiece, in which residual cytoplasm and mitochondria can be found (24). Kamal et al., reported a negative association between seminal clusterin (involved in the cellular debris clearance and apoptosis) gene expression and sperm motility, concentration, and acrosin activity, linearity index and velocity, and a positive association with sperm DNA fragmentation and abnormal forms (25). Roshdy and Mostafa reported an association between survivin protein (an apoptosis inhibitor) in the seminal plasma of fertile and infertile males and sperm motility processes and spermatogenesis (26). These results are consistent with our findings regarding the interdependence between the expression of genes involved in apoptosis (*BAX, BCL-2*), and decreased sperm quality.

The current study describes the correlation between apoptosis markers such as *BAX*, and *BCL-2 *mRNA expression with fertilization rate, cleavage rate, and embryo quality in oligoasthenoteratozoospermic men under ICSI processing. Based on this study, changes in the expression of *BAX*, and *BCL-2* genes indirectly through reducing the sperm quality and increasing the sperm DNA damage level might compromise the progression of embryo development, resulting in arrested embryos. Sperm DNA integrity is of great importance as the genetic material quality in a sperm population is important for the subsequent and embryonic development in children, successful fertilization, and reproductive potential in males. Apoptosis is necessary for male gametes from the gonadal anlagen appearance in the embryo to fertilization. It can maintain the proper ratio between the Sertoli cells and germ cells during prenatal development (27).

Apoptosis removes damaged cells from the testicles in the samples of adults. Mature spermatozoa experience apoptosis and phagocytosis in the reproductive tract of females to avoid the inflammatory reaction due to the dead gamete destruction (28). The apoptosis impairment leads to developmental abnormalities in infertility, gametes of males, and oocyte fertilization by sperm with DNA damage leading to ART failure (29). Thus, apoptosis is an important cause of sperm DNA fragmentation, leading to poor embryo quality at day 3, slower rate of cleavage, poor blastocyst development, and implantation for the widespread use of assisted reproductive technologies (30). On the other hand, apoptosis shows a significant correlation with sperm morphology. Sperm head impairments are linked to the last phases of embryonic development, sperm midpiece impairments to intermediate cleaving embryos, and tail impairments to the initial embryonic development phases (31). In fact, in this study, we evaluated the sperm *BCL-2* and *BAX* can affect the quality of the embryo through the effect on sperm morphology. Further studies are needed to confirm this hypothesis.

## 5. Conclusion 

Moreover, according to the results, there is a relationship between apoptosis gene transcript levels sperm quality, and clinical outcomes in the oligoasthenoteratozoospermic group. However, more studies with a larger series are needed to be sure that these genes can influence reproductive outcomes.

##  Data availability

The primary data for this study is available from the authors upon direct request.

##  Author contributions

R.J., F.B., and M.Kh.M., were involved in the conception, design, statistical analysis, and drafting of the manuscript. All authors have approved the final version for submission.

##  Conflict of Interest

The authors declare that there is no conflict of interest.
